# Supplementary material for: Costs of implementing universal test and treat in three correctional facilities in South Africa and Zambia
Source: PLoS One. 2022 Aug 25;17(8):e0272595. doi: 10.1371/journal.pone.0272595 (PMC9409581; doi:10.1371/journal.pone.0272595)
Supplement: S1 Table — (DOCX) [file pone.0272595.s001.docx]

S1 Table. Service statistics over a 12-month period at correctional facilities in 2017

| Service statistics | Brandvlei Correctional | Johannesburg  Correctional | Lusaka  Central |
| --- | --- | --- | --- |
| Clients pre-test counselled | 1 901 | 3 059 | 2 481 |
| Clients tested for HIV | 1 901 | 3 059 | 2 481 |
| Clients tested HIV Positive | 42 | 448 | 435 |
| Clients initiated on ART | 35 | 233 | 229 |
| Clients TB symptom screened | 36 | 130 | 222 |
| Clients maintained on ART | 22 | 144 | 82 |
|  |  |  |  |
