# Supplementary material for: Costs of implementing universal test and treat in three correctional facilities in South Africa and Zambia
Source: PLoS One. 2022 Aug 25;17(8):e0272595. doi: 10.1371/journal.pone.0272595 (PMC9409581; doi:10.1371/journal.pone.0272595)
Supplement: S2 Table — (DOCX) [file pone.0272595.s002.docx]

S2 Table. Equipment lists for Brandvlei, Johannesburg and Lusaka correctional facilities

| **Item - Brandvlei and Johannesburg Correctional** | **Item - Lusaka Central** |
| --- | --- |
| Biohazard Box  Cell phone (Pre-paid)  Chairs  Cooler Box  Counselling Flipchart  Desk  Filing cabinets  Fridge  Laptop / desktop computer  Perforator  Physical Exam Equipment  Blood Pressure Machine  Exam Table  Otoscope  Stethoscope  Thermometer  Torch  Weighing Scale  Portable CD reader  Stapler/Staples  Storage Boxes  Table  Tourniquet  Water basin/Sink  Xpert Machine | Cell phone (Pre-paid)  Chairs  Cooler Box  Counselling Flipchart  Desk  Filing cabinets  Fridge  Laptop / desktop computer  Perforator  Physical Exam Equipment  Blood Pressure Machine  Exam Table  Otoscope  Stethoscope  Thermometer  Torch  Weighing Scale  Portable CD reader  Stapler/Staples  Storage Boxes  Table  Water basin/Sink  Xpert Machine  Biohazard Box  Tourniquet |
