# Supplementary material for: Costs of implementing universal test and treat in three correctional facilities in South Africa and Zambia
Source: PLoS One. 2022 Aug 25;17(8):e0272595. doi: 10.1371/journal.pone.0272595 (PMC9409581; doi:10.1371/journal.pone.0272595)
Supplement: S3 Table — (DOCX) [file pone.0272595.s003.docx]

S3 Table. Supplies lists for Brandvlei, Johannesburg and Lusaka correctional facilities

| **Item - Brandvlei and Johannesburg Correctional** | **Item – Lusaka Central** |
| --- | --- |
| A4 book  Admission Book  Airtime  Alcohol swabs/webcol  Antiretroviral Therapy  Anti-Tuberculosis Therapy  Biohazard Bags  Biohazard Box  Cleaning supplies  Clinical Forms  Blood Collection Tube  Vacutainer Needle  Vacutainer Needle Holder  Cotton Wool  Alcohol Swabs  Band-Aids  Lab Forms  Tourniquet  Sputum Bottle  Specimen Bags  Cotrimoxazole  Cotton Roll  Fasteners/Folders/Hanging Folders  Fluconazole  Gloves  Inkpad/Ink  Lab Forms  Methylated Spirit  Pamphlets  Proficiency testing panel  Register (HTC Register, Daily Register, TB Register)  Screening tool (HTS)  Sharps Container  Sputum Book/TB Suspect Register  Oraquick Advance Rapid HIV 1/2 Antibody  Alere Determine HBV Test Kit  Abon HIV Tri-line Rapid Tests  Alcohol Swabs  Cotton Wool  Gloves  Hand Sanitizer  Linen Saver  Medical Handwash  Results Slips/Envelopes  Tongue Depressor  Urine dipstick  Xpert Cartridges, Buffer & Supplies | A4 book  Admission Book  Airtime  Alcohol swabs/webcol  Antiretroviral Therapy  Anti-Tuberculosis Therapy  Biohazard Bags  Biohazard Box  Cleaning supplies  Clinical Forms  Blood Collection Tube  Vacutainer Needle  Vacutainer Needle Holder  Cotton Wool  Alcohol Swabs  Band-Aids  Lab Forms  Tourniquet  Sputum Bottle  Specimen Bags  Cotrimoxazole  Cotton Roll  Fasteners/Folders/Hanging Folders (each)  Fluconazole  Gloves  Inkpad/Ink  Lab Forms  Methylated Spirit  Pamphlets (each)  Proficiency testing panel  Register (HTC Register, Daily Register, TB Register)  Screening tool (HTS)  Sharps Container  Sputum Book/TB Suspect Register  Determine  Bioline  Alere Determine HBV Test Kit 100 test  Alcohol Swabs  Cotton Wool  Gloves  Hand Sanitizer  Linen Saver (Pack of 10)  Medical Handwash  Results Slips/Envelopes  Tongue Depressor Per 100  Urine dipstick  Xpert Cartridges, Buffer & Supplies |
